# Self-organising aggregates of zebrafish retinal cells for investigating mechanisms of neural lamination

**DOI:** 10.1242/dev.142760

**Published:** 2017-03-15

**Authors:** Megan K. Eldred, Mark Charlton-Perkins, Leila Muresan, William A. Harris

**Affiliations:** Department of Physiology, Development and Neuroscience, Cambridge University, Cambridge CB2 3DY, UK

**Keywords:** Müller cells, Cell sorting, Layer formation, Organoid, Reaggregation, SoFa

## Abstract

To investigate the cell-cell interactions necessary for the formation of retinal layers, we cultured dissociated zebrafish retinal progenitors in agarose microwells. Within these wells, the cells re-aggregated within hours, forming tight retinal organoids. Using a Spectrum of Fates zebrafish line, in which all different types of retinal neurons show distinct fluorescent spectra, we found that by 48 h in culture, the retinal organoids acquire a distinct spatial organisation, i.e. they became coarsely but clearly laminated. Retinal pigment epithelium cells were in the centre, photoreceptors and bipolar cells were next most central and amacrine cells and retinal ganglion cells were on the outside. Image analysis allowed us to derive quantitative measures of lamination, which we then used to find that Müller glia, but not RPE cells, are essential for this process.

## Abstract (precis)

**Summary:** Müller glia cells, but not retinal pigmented epithelial cells, are crucial for retinal cell lamination in retinal organoids grown *in vitro*.

## INTRODUCTION

The retina is a strikingly well-organised neural tissue, with each of the major cell types sitting in its own specific layer. Such laminated cellular organisation, which is common in the nervous system, may aid in wiring the brain efficiently during development. However, the mechanisms involved in the development of lamination, are only beginning to be understood. In the cerebral cortex, there is a well-known histogenetic organisation, with early born cells populating the deep layers and late-born cells populating the superficial layers, an ‘inside-out’ order (McConnell, 1995). But timing alone does not account for this organisation, as is clearly shown in the example of *reeler* mutant mice, where the neocortex, shows the opposite ‘outside-in’ order of histogenesis, even though the different types of cortical cells are generated and migrate to the cortical plate at the correct times (Caviness and Sidman, 1973). The layering defect in *reeler* is due to the lack of the glycoprotein reelin, which is secreted largely by a single transient cell type, the Cajal-Retzius cell (D'Arcangelo and Curran, 1998; Huang, 2009), suggesting certain cells and molecules play important roles in histogenesis.

Retinal cells, like cells of the cerebral cortex, show a histogenetic arrangement, with early born retinal ganglion cells (RGCs) residing in the innermost retinal layer and late-born photoreceptors in the outermost retinal layer (Cepko et al., 1996; Harris, 1997). But again, the mechanism here cannot simply be timing – i.e. cells piling up on top of each other according to their birthdate. This is known because several studies have revealed that the different retinal cell types are born with overlapping periods of birth, suggesting that timing alone is insufficient (Holt et al., 1988). In zebrafish, live imaging studies have revealed that sister cells born at exactly the same time may migrate to different but appropriate layers (He et al., 2012), that late-born RGCs migrate through earlier born amacrine cells (ACs) to reach the RGC layer, and that there is a period during which postmitotic cells intermingle before they sort into their correct layers (Almeida et al., 2014; Chow et al., 2015). One issue arising from these findings is whether these behaviours result from interactions between the different cell types, i.e. cell-cell interactions, or from different cell types responding to common environmental cues, such as gradients of apicobasal cues. The latter possibility is consistent with *in vivo* studies in which lamination is preserved even in the absence of specific cell types (Green et al., 2003; Kay et al., 2004; Randlett et al., 2013). However, other studies suggest that direct interactions between cell types are likely to be involved in normal layering (Huberman et al., 2010; Chow et al., 2015). In addition, the involvement of cell-cell interactions is indicated by the formation of rosettes in retinoblastoma (Johnson et al., 2007) and retinal dysplasias in which cell adhesion molecules such as N-cadherin are compromised (Wei et al., 2006).

Aggregation cultures, used since the early 20th century have revealed the ability of various cell types to re-aggregate and re-organise into histotypic tissues in the absence of tissue scaffolds and extrinsic factors. This phenomenon was first seen in basic, monotypic tissues, such as sponge and sea urchin (Herbst, 1900; Wilson, 1907), not only revealing an innate ability of certain cell types to self-organise, but also providing a platform on which we could begin to investigate the fundamental cell-cell interactions involved in histogenesis. In the mid-century, Moscona and colleagues used aggregation studies to investigate tissue formation in a variety of tissues, including the chick retina (Moscona and Moscona, 1952; Moscona, 1961), highlighting the ability of even complex, multitypic tissues to self-organise. Later, Layer and colleagues were able to generate fully stratified retinal aggregates, termed retinospheroids, from embryonic chick retinal cells in rotary culture (Layer and Willbold, 1993, 1994; Rothermel et al., 1997). The study of aggregation cultures has led to physical and theoretical considerations of how tissues might self-organise, including differential adhesion or tension between cells (Steinberg, 2007; Heisenberg and Bellaïche, 2013).

In this paper, we present the embryonic zebrafish retina as a model with which to extend these investigations due to the increasing availability of genetic, molecular and nanophysical tools with which to label and manipulate cells types and molecules of interest. We use the transgenic Spectrum of Fates (SoFa1) fish, in which all retinal cell types are labelled, in aggregate cultures to examine the ability of zebrafish retinal cells to self-organise, and we investigate the importance of retinal pigment epithelial cells and Müller cells in retinal lamination.

## RESULTS

### Dissection, dissociation and culture of zebrafish retinal cells

**Fig. 1.**
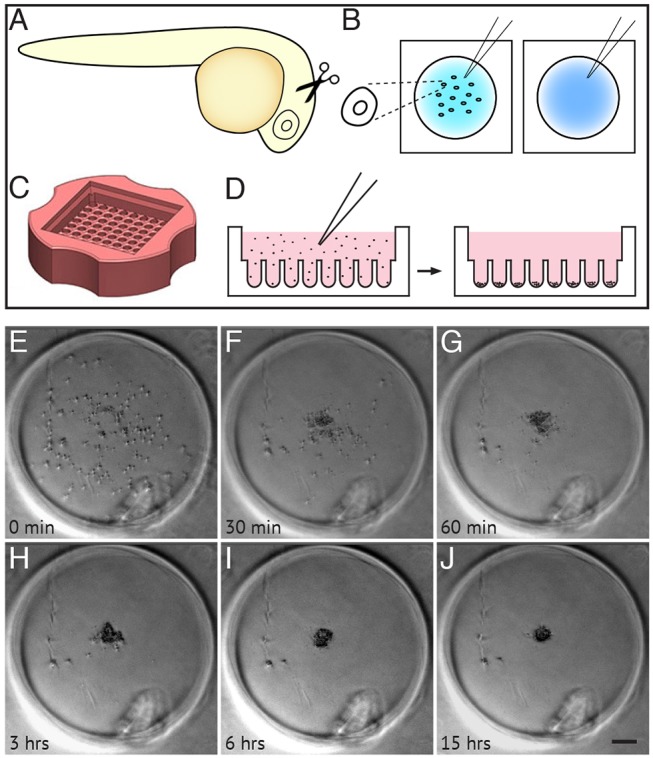
**Dissociation, culture and re-aggregation of zebrafish retinal cells.** (A,B) Schematic representing retinas dissected from 24 hpf zebrafish (A), collected into glass dishes and dissociated into single cells (B). (C) Agarose microwell dish cast from the 3D Petri Dish PDMS Mould (adapted, with permission, from http://www.microtissues.com). (D) Schematic representing the seeding chamber of the 3D Petri dish. After seeding, cells settle into individual wells. (E-J) Time-lapse images of a single well from the 3D Petri dish showing 24 hpf cells re-aggregating. (H) Cells are almost fully reaggregated 3 h after seeding. (J) Cells have undergone compaction 15 h after seeding. Time is in minutes and hours after seeding. Scale bar: 100 μm.

At 24 h post fertilisation (hpf), the zebrafish retina is a pseudostratified epithelium comprising ∼2000 progenitor cells, each stretching from the apical to the basal surface. Over the next 48 h, these progenitors divide several times to give rise to a fully laminated retina of ∼22,000 postmitotic neurons and glia of all the major cell types (He et al., 2012). We dissected and dissociated retinas within this time window in order to investigate the cell interactions at these times (Fig. 1A,B). To assure ourselves that the dissociation protocol was satisfactory, we used a fluorescent cell counter (see Materials and Methods) to assess several factors. Cell yield was consistently high, between 2000 and 3000 cells per 24 hpf retina (Fig. S1A); cluster analysis showed that over 95% of these dissociated cells were counted as single cells (Fig. S1B); and cell viability immediately after dissociation was over 96%, as calculated using the Acridine Orange/Propidium Iodide viability assay (Fig. S1C). With sufficient cell yield and viability, we began our reaggregation experiments in a basic L-15 supplemented with PSF, but found that the addition of zebrafish embryo extract and fetal bovine serum promotes cell re-aggregation and growth (Fig. S1D-G). In agreement with previous reports (Zolessi et al., 2006), we also found that N2 supplement supports RGC growth and maturation in these cultures (data not shown).

To investigate the cell-cell interactions involved in layering, we wanted to reaggregate the cells in a way that minimises interactions with the substrate, thus limiting all interactions to those between the cells themselves. For this reason, we tried a traditional hanging-drop culture (Foty, 2011). We seeded aliquots of the single cell suspension in drops on the lids of culture dishes, which were then inverted (Fig. S1H). After 48 h, we found varying degrees of aggregation; some drops contained single large clusters whereas others contained several smaller clusters (Fig. S1I). We obtained much more consistent results when we plated the dissociated cells into agarose microwells made using the 3D petri dish mould (Napolitano et al., 2007; Klopper et al., 2010) (Microtissues) (Fig. 1C,D; Fig. S1J,K). These agarose microwells provide a confined, non-adhesive environment that minimises distances between cells. The dissociated cells in these wells began to aggregate immediately after seeding. Within 3 h most cells had aggregated (Movie 1), and by 15 h the cells had undergone compaction into similarly sized aggregates (Fig. 1E-J).

The ability of zebrafish retinal progenitors to reaggregate without the need for a scaffold supports previous findings in chick from the Moscona laboratory (Moscona, 1961; Sheffield and Moscona, 1969). In those studies, they identified a cell reaggregation-promoting factor (Lilien and Moscona, 1967), which was later cloned and identified as retinal cognin (R-Cognin) (Hausman and Moscona, 1976). To assess whether the same factor was involved in the reaggregation of zebrafish retinal cells, we added PACMA31, a small molecule inhibitor of the active site of R-Cognin, to our cultures. We found a dose-dependent effect on aggregations: cells treated with 5 μM of PACMA31 generated slightly loose aggregates after 24 h in culture, whereas those treated with 50-200 μM were completely unable to aggregate (Fig. S2).

### A self-organising retina: identification of zebrafish retinal cells and characterisation of organisation

**Fig. 2.**
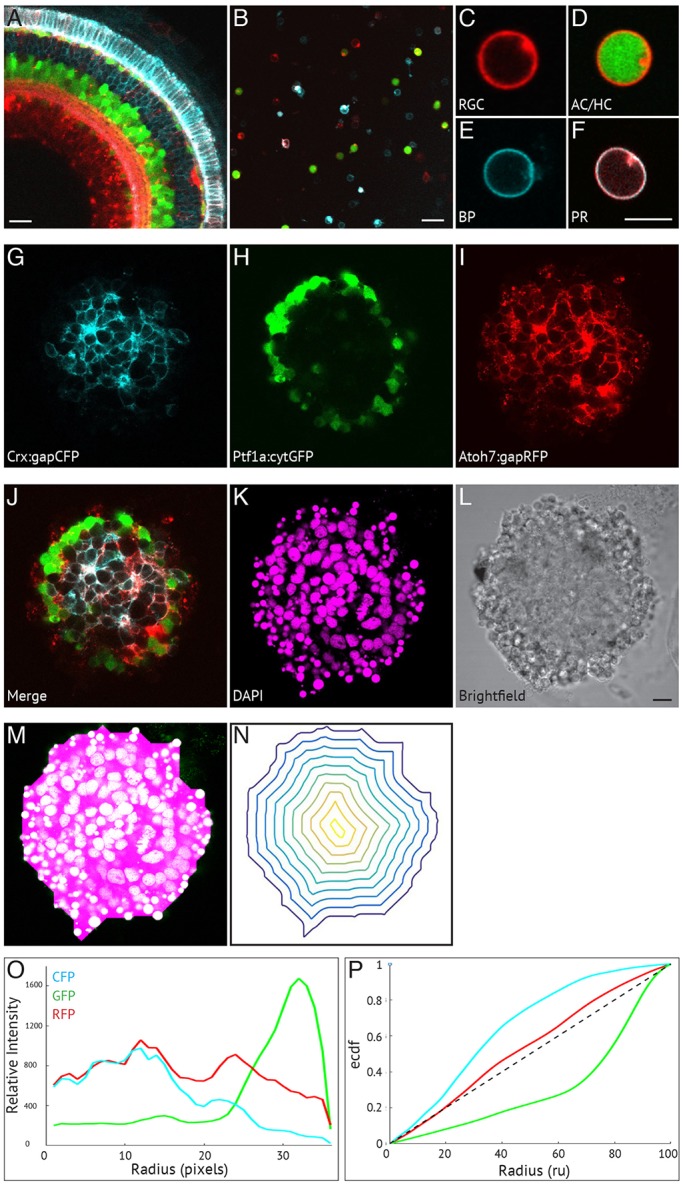
**A self-organising retina: identification of zebrafish retinal cells and characterisation of organisation.** The main cell types of the retina can be identified in the SoFa1 transgenic line (Almeida et al., 2014) using a combination of genetically tagged cell fate markers: Atoh7:gapRFP labels RGC, AC/HC and PR cell membranes; Ptf1a:cytGFP labels AC/HC cytoplasm; and Crx:gapCFP labels BP and PR membranes. (A) Central sagittal section of a region of the SoFa1 retina. Scale bar: 20 μm. (B) Dissociated cells of the SoFa1 line. Scale bar: 20 μm. (C-F) Individual cells are identified based on their spectral expression: (C) RGCs express membrane RFP; (D) AC/HCs express cytoplasmic GFP and membrane RFP; (E) BPs express membrane CFP; and (F) PRs express membrane CFP and RFP. Scale bar: 5 μm. (G-L) Central sagittal section of a retinal aggregate cultured using the SoFa1 line. (G) Crx:gapCFP-expressing cells are found in the centre of the aggregate. (H) Ptf1a:cytGFP-expressing cells are found in a distinct ring around the Crx:gapCFP population. (I) Atoh7:gapRFP-expressing cells are found throughout the aggregate. (J) Merge of channels represented in G-I. (K) DAPI. (L) Bright-field image. Scale bar: 10 μm. (M-P) Generation of analysis of cellular organisation using custom-made Matlab scripts. (M) A mask is fitted to the aggregate using the DAPI channel. (N) Successive isocontours are fitted from the periphery to the centre of the aggregate. (O) Fluorescence is measured along each contour and plotted as a relative fluorescence intensity (*y*-axis) against radial position (in pixels) (*x*-axis). (P) Fluorescence profiles for each channel are plotted as an empirical cumulative distribution function (ECDF) (*y*-axis) against radial position [radial units (ru)] (*x*-axis). The dotted diagonal line represents a theoretically perfect even distribution of fluorescence from centre to periphery.

The Spectrum of Fates (SoFa1) zebrafish transgenic line (Almeida et al., 2014) allows the simultaneous identification of all five main retinal cell types based on three fluorophores, each of which is expressed in particular combinations of retinal cell types (Fig. 2A-F). RGCs express membrane-bound RFP (Fig. 2C); amacrine and horizontal cells (ACs and HCs) express cytoplasmic GFP and membrane-bound RFP (Fig. 2D); bipolar cells express membrane-bound CFP (Fig. 2E); and photoreceptors express membrane-bound CFP and RFP (Fig. 2F). Whereas most studies of tissue organisation use techniques such as immunohistochemistry or *in situ* hybridisation to identify the different cell populations, the use of SoFa1 line as the starting material for these studies allows immediate and even live microscopic access to the process of lamination.

As was previously reported in the studies of chick retinal reaggregation assays (Rothermel et al., 1997), we also found that the developmental stage of the cells when they are dissociated and re-aggregated has an effect on their ability to organise. Cultures from cells of younger stage embryos (e.g. 24 hpf) are more capable of organising than those from older stages (e.g. 72 hpf) (Fig. S3), suggesting the mechanisms responsible for retinal layering are active during the developmental stages when these processes are normally occurring.

Using this strategy, we found that aggregates derived from 24 hpf zebrafish retinal progenitors are indeed capable of self-organising. Fig. 2G-L shows the central sagittal section of an aggregated retinal culture after 48 h in culture. It can be seen quite clearly that the Ptf1a:cytGFP-expressing cells (ACs and HCs) organise in a distinct ring near the outside of the aggregate (Fig. 2H), containing within them a cluster of Crx:gapCFP-expressing cells (PRs and BCs) (Fig. 2G). It is difficult to see the positioning of RGCs in this preparation as Atoh7:gapRFP is expressed in many other cells types; however, Zn5 antibody staining reveals RGCs positioned in the outer layer of the aggregate, among the Ptf1a:cytGFP cells (Fig. S4). The organisation of these aggregates appears to be ‘inside-out’ with respect to the normal retina. Thus, although situated near the basement membrane on the inner surface of the intact retina, RGCs in our aggregates are found near the outer surface, and photoreceptors and bipolar cells, which populate the outer layers of the intact retina, are found near the centres of our aggregates. To assess whether this organisation was similar to that in the intact eye in terms of cell numbers, we counted the relative proportions of cell types in our aggregate cultures by counting the numbers in each fluorescent channel as a proportion of total cells. We found the numbers of ACs and HCs to be very similar to those in previously published *in vivo* studies (Boije et al., 2015; He et al., 2012), whereas the numbers of BCs and PRs were somewhat increased (Table S1). The reason for this is unknown, but the overall change in proportions is fairly modest. Therefore, perhaps it is not unreasonable to find that the organisation seen in our aggregates resembles the situation *in vivo*.

### Quantification

This pattern of organisation clearly shows relative positions of cell types in our aggregates, as reflected in the fluorescence profiles, which are highly consistent within and between experiments, making it a good platform from which to compare experimental conditions. To begin to quantitate this pattern, we devised a Matlab script, which generated an isocontour fluorescence profile for each aggregate. This fits a mask to the aggregate (Fig. 2M) and isocontours from the periphery to the centre of the aggregate (Fig. 2N), along which it gives a readout of the fluorescence distribution across isocontours of the distance function from the outline of the aggregate for each fluorescent protein (FP) (for further details, see Materials and Methods). Fig. 2O shows the fluorescence profile for the aggregate represented in Fig. 2G-L. The CFP expression is high near the centre of the aggregate, tailing off towards the periphery, whereas the GFP expression is low in the centre, but peaks near the periphery, corresponding roughly with Crx:gapCFP and Ptf1a:cytGFP cell positions, respectively. By plotting these data as an empirical cumulative distribution function (ECDF) against radial position, we are able to see how far these patterns of expression deviate from a random distribution of expression, which would be a straight diagonal line from the bottom left to the top right. Fig. 2P shows that the distribution of Atoh7:gapRFP curve is close to such a straight line (dotted line). This is due to the fact that Atoh7 is expressed in most of the different cell types, indicating an even patterning of that fluorescent marker across the aggregate, consistent with a complete failure of patterning. The ECDF for Crx:gapCFP-expressing cells is clearly shifted to the left of this line, whereas distribution of Ptf1a:cytGFP cells is shifted to the right. By measuring the areas between these curves, we can derive a measure of laminar organisation in our organoids, and can easily compare one experiment with another.

### RPE is not required for self-organisation

With the experimental and analytical tools in hand, we moved our focus to the mechanisms responsible for this organisation. One approach to investigate these is to eliminate specific cell types to see whether any particular cell type is required. Previous studies in chick have pointed to the retinal pigment epithelium (RPE) as being important for retinal organisation by providing polarity information. Chick retinal cells cultured in the absence of RPE formed aggregates containing rosettes with inverted layering, but when cultured in the presence of a monolayer of RPE, formed correctly oriented fully stratified retinospheroids (Rothermel et al., 1997).

**Fig. 3.**
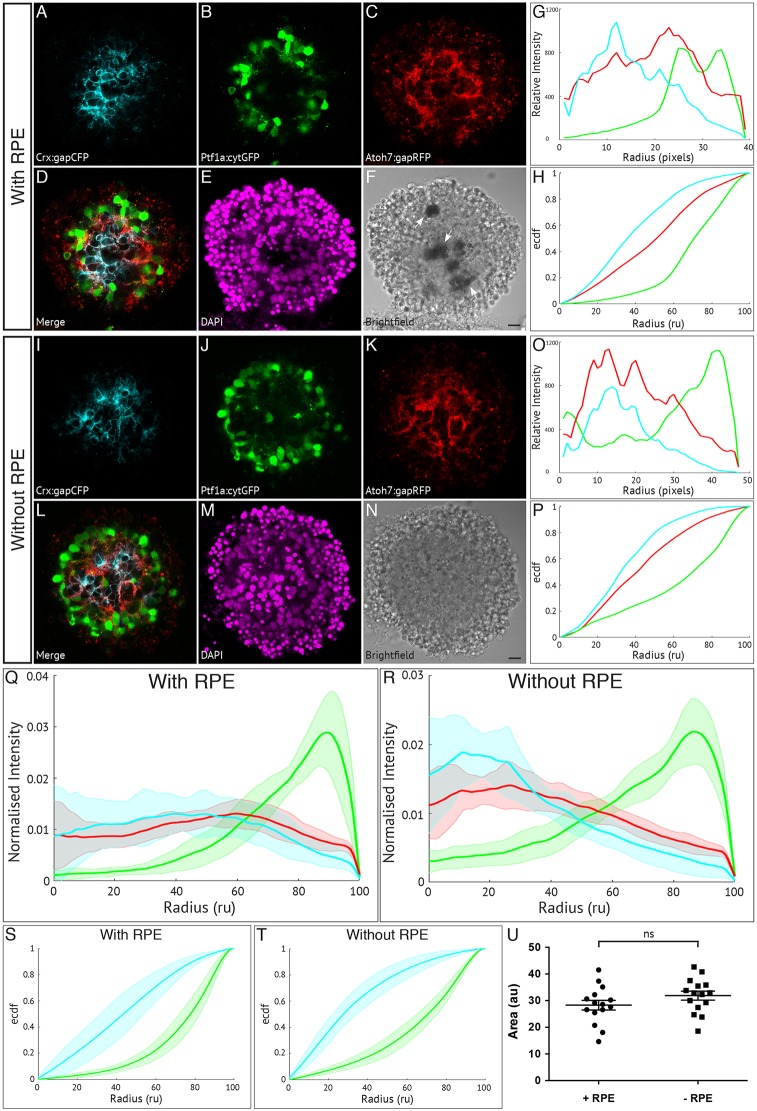
**Retinal pigment epithelium is not required for zebrafish retinal self-organisation.** Fluorescence profiles are generated for SoFa1 aggregates cultured either with or without RPE cells. (A-F) Central sagittal section of a SoFa1 aggregate with RPE. (A) Crx:gapCFP-expressing cells are found in the centre of the aggregate. (B) Ptf1a:cytGFP-expressing cells are found in a ring around the edge of the Crx:gapCFP population. (C) Atoh7:gapRFP-expressing cells are found throughout the aggregate. (D) Merge of channels represented in (A-C). (E) DAPI. (F) Bright-field image. Pigment-expressing RPE cells can be seen near the centre of the aggregate (arrows). Scale bar: 10 μm. (G) Fluorescence profiles for the aggregate represented in A-F. (H) ECDF plot for the aggregate represented in A-F. (I-N) Central sagittal section of a SoFa1 aggregate without RPE. (I) Crx:gapCFP-expressing cells are found in the centre of the aggregate. (J) Ptf1a:cytGFP-expressing cells are found in a ring around the edge of the Crx:gapCFP population. (K) Atoh7:gapRFP-expressing cells are found throughout the aggregate. (L) Merge of channels represented in I-K. (M) DAPI. (N) Bright-field image. No pigment-expressing RPE cells can be seen. Scale bar: 10 μm. (O) Fluorescence profiles for the aggregate represented in I-N. (P) ECDF plot for the aggregate represented in I-N. (Q) Average fluorescence profiles with shaded error for aggregates with RPE (*n*=15, three experimental repeats). (R) Average fluorescence profiles with shaded error for aggregates without RPE (*n*=15, three experimental repeats). (S) Average ECDF plots for aggregates with RPE. (T) Average ECDF plots for aggregates without RPE. (U) Area (in arbitrary units) is calculated between the ECDF for the Crx:gapCFP population and the ECDF for the Ptf1a:cytGFP population of cells, and compared between aggregates with RPE (+RPE) and without RPE (–RPE) (*n*=15 for each condition, Mann–Whitney two-tailed *t*-test, *P*>0.05).

We therefore made reaggregates with and without RPE. RPE cells were included (Fig. 3A-H) or excluded (Fig. 3I-P) either by gently removing the layer during dissection, or by leaving the layer attached to the neural retina before dissociation. These experiments were carried out using 32 hpf embryos to allow us to identify RPE cells based on pigment formation, while retaining a similar level of organisation to those from 24 hpf (Fig. S3A-J). It is clear that the fluorescence profiles of cultures with RPE (Fig. 3G) and without RPE (Fig. 3O) are in the same order, with the Crx:gapCFP profile peaking towards the centre of the aggregate and the Ptf1a:cytGFP profile peaking towards the periphery. This pattern is consistent across all aggregates analysed (Fig. 3Q,R). This is also represented in the ECDF plots in which for aggregates with RPE (Fig. 3H) and without RPE (Fig. 3P) the Crx:gapCFP curve is shifted to the left of the Atoh7:gapRFP curve, and the Ptf1a:cytGFP curve is shifted to the right. The somewhat different shapes of the curves near the centre of the aggregate for the condition with RPE is due to the fact that the pigment epithelial cells, which are themselves not fluorescent, are positioned close to the centre of these aggregates. Areas measured between these curves for both conditions show no significant difference (Fig. 3S-U). These results, together with the fact that in both conditions the aggregates show a similar degree of ordering in the same relative patterns, suggest that in these experiments, RPE cells may not have an appreciable influence on the ability of developing retinal tissue to self-organise.

### Müller glia are important for retinal cell organisation

**Fig. 4.**
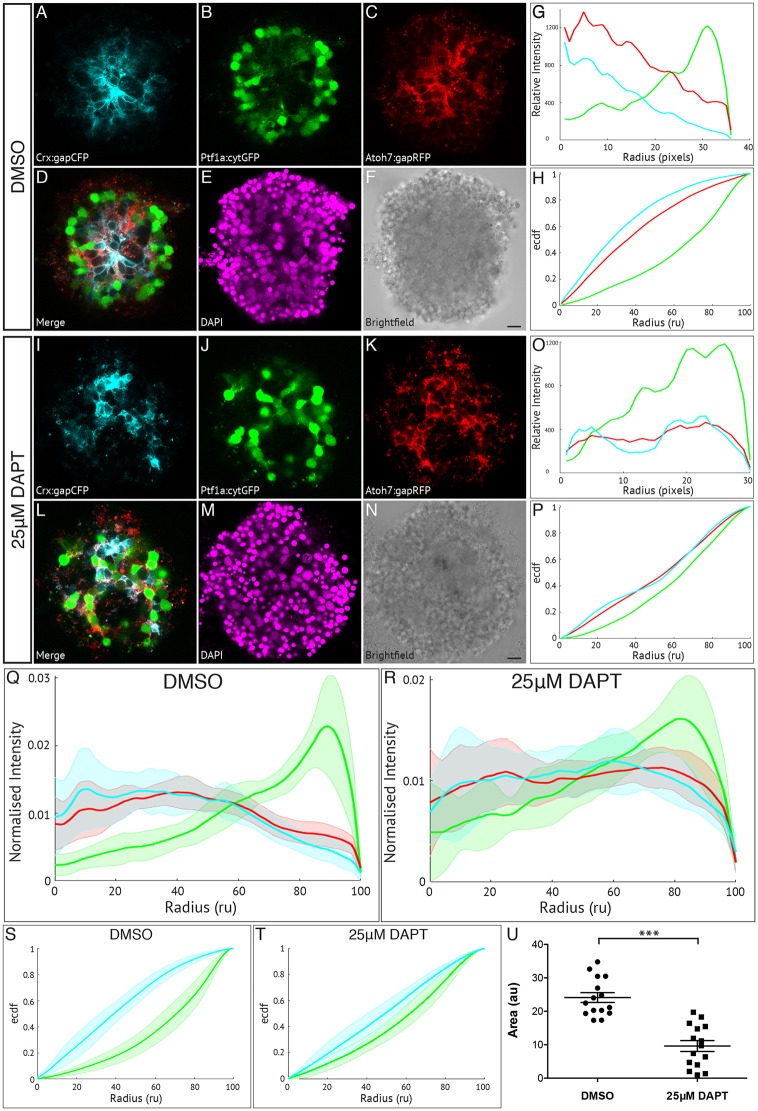
**Müller glia are important in zebrafish retinal self-organisation.** Fluorescence profiles are generated for SoFa1 aggregates treated either with 25 μM DAPT to prevent the differentiation of Müller glia or with DMSO as a control. (A-F) Central sagittal section of a SoFa1 aggregate treated with DMSO. (A) Crx:gapCFP-expressing cells are found in the centre of the aggregate. (B) Ptf1a:cytGFP-expressing cells are found in a ring around the edge of the Crx:gapCFP population. (C) Atoh7:gapRFP-expressing cells are found throughout the aggregate. (D) Merge of channels represented in A-C. (E) DAPI. (F) Bright-field image. Scale bar: 10 μm. (G) Fluorescence profiles for the aggregate represented in A-F. (H) ECDF plot for the aggregate represented in A-F. (I-N) Central sagittal section of a SoFa1 aggregate treated with 25 μM DAPT. (I) Some Crx:gapCFP-expressing cells are found in the centre of the aggregate, and some are found nearer the edge. (J) Ptf1a:cytGFP-expressing cells are found throughout the aggregate. (K) Atoh7:gapRFP-expressing cells are found throughout the aggregate. (L) Merge of channels presented in I-K. (M) DAPI. (N) Bright-field image. Scale bar: 10 μm. (O) Fluorescence profiles for the aggregate represented in I-N. (P) ECDF plot for the aggregate represented in I-N. (Q) Average fluorescence profiles with shaded error for aggregates treated with DMSO (*n*=15, three experimental repeats). (R) Average fluorescence profiles with shaded error for aggregates treated with 25 μM DAPT (*n*=15, three experimental repeats). (S) Average ECDF plots for aggregates treated with DMSO. (T) Average ECDF plots for aggregates treated with 25 μM DAPT. (U) Area (in arbitrary units) is calculated between the ECDF for the Crx:gapCFP population and the ECDF for the Ptf1a:cytGFP population of cells, and compared between aggregates treated with DMSO and aggregates treated with 25 μM DAPT (*n*=15 for each condition, Mann–Whitney two-tailed *t*-test, *P*<0.0001).

We next tested whether Müller glia have a role in the lamination of our retinal organoids. Importantly, we found that Müller cell numbers are similar in our aggregates compared with those counted *in vivo* (Table S1). Müller glia cells were eliminated by treatment with the Notch inhibitor DAPT, which was applied to our cultures from the time equivalent of 45-48 hpf in the embryo onwards. Treatment of embryos at this time completely blocks the differentiation of Müller glia *in vivo* without affecting the differentiation of any of the neural cell types (MacDonald et al., 2015). The GFAP:GFP reporter line (Bernardos and Raymond, 2006) was used to confirm the presence or absence of Müller glia in our aggregates (Fig. S5). DMSO-treated controls show a high expression of GFAP:GFP, with Müller glia extending processes throughout the aggregate (Fig. S5A), whereas aggregates treated with 25 μM DAPT display vastly reduced expression of GFAP:GFP and no process projections (Fig. S5C). We then analysed the effect of removing Müller glia on the ability of all other cell types to organise using the SoFa1 line. The morphology of the aggregates (Fig. 4A-F) and fluorescence profiles of DMSO-treated aggregates (Fig. 4G) are similar to previous control aggregates, with the Crx:gapCFP profile peaking towards the centre of the aggregate and the Ptf1a:cytGFP profile peaking towards the periphery. This is consistent across all aggregates analysed for this condition (Fig. 4Q). This is also represented in the ECDF plot (Fig. 4H) where the Crx:gapCFP curve is shifted to the left of the Atoh7:gapRFP curve, and the Ptf1a:cytGFP curve is shifted to the right. The DAPT-treated cultures show disorganised aggregates (Fig. 4I-N) and the corresponding fluorescence profiles clearly differ from the controls (Fig. 4O); the lack of pattern is seen in all aggregates analysed for this condition (Fig. 4R). The Crx:gapCFP curve does not peak in the centre of the aggregate, but rather shows more of a plateau, with two smaller peaks (one nearer the centre and one nearer the periphery), while the Ptf1a:cytGFP profile still peaks towards the periphery but the gradient is much reduced. These trends are reflected in the ECDF plots for the DAPT-treated culture, where it is clear that both the Crx:gapCFP and the Ptf1a:cytGFP have both been shifted towards the Atoh7:gapRFP curve (Fig. 4P), representing an almost complete failure of patterning. Areas measured between these curves for both conditions show a significantly higher order of organisation for the DMSO-treated controls as compared with the DAPT-treated cultures (Fig. 4S-U). These results suggest that Müller glia may play an important role in the laminar organisation of retinal organoids.

**Fig. 5.**
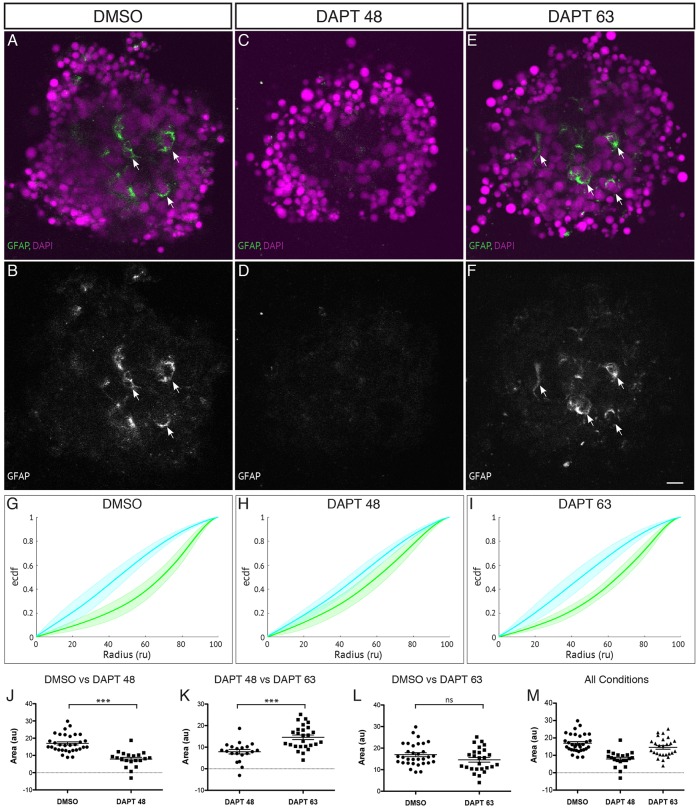
**Late application of DAPT allows Müller glia to be generated and aggregates to self-organise.** Aggregates are cultured in the presence of DAPT applied at 45-48 hpf onwards to block the differentiation of Müller glia or at 63 hpf onwards to allow the differentiation of some Müller glia compared with DMSO control. (A,B) Aggregates cultured in the presence of DMSO show several GFAP-positive cells (indicated by arrows). (C,D) Aggregates cultured in the presence of DAPT from 45-48 hpf onwards have little or no GFAP-positive cells. (E,F) Aggregates cultured in the presence of DAPT from 63 hpf onwards have several GFAP-positive cells (indicated by arrows). Scale bar: 10μm. (G) Average ECDF plots for aggregates treated with DMSO. (H) Average ECDF plots for aggregates treated with DAPT from 45-48 hpf onwards. (I) Average ECDF plots for aggregates treated with DAPT from 63 hpf onwards. (J) Area (in arbitrary units) is calculated between the ECDF for the Crx:gapCFP population and the ECDF for the Ptf1a:cytGFP population of cells, and compared between aggregates treated with DMSO and aggregates treated with DAPT from 45-48 hpf onwards (*n*=32 for DMSO, *n*=20 for DAPT at 45-48 hpf, Mann–Whitney two-tailed *t*-test, *P*<0.0001). (K) Area (arbitrary units) compared between the ECDF plots of aggregates treated with DAPT from 45-48 hpf onwards and aggregates treated with DAPT from 63 hpf onwards (*n*=20 for DAPT at 45-48 hpf, *n*=26 for DAPT at 63 hpf, Mann–Whitney two-tailed *t*-test, *P*<0.0001). (L) Area (arbitrary units) compared between the ECDF plots of aggregates treated with DMSO and aggregates treated with DAPT from 63 hpf onwards (*n*=32 for DMSO, *n*=26 for DAPT at 63 hpf, Mann–Whitney two-tailed *t*-test, *P*>0.05). (M) Areas of data from all conditions.

To address the issue of whether this phenotype may be due to effects of inhibiting Notch during the later stages of organisation, or due to an alternative effect of inhibiting gamma secretase activity, we carried out further experiments where we applied DAPT to our cultures at a later time point to allow some Müller glia to differentiate. First, we showed that Müller cells arise at approximately the same time in organoids as they do *in vivo* (Fig. S6). Aggregates in which DAPT was added at 63 hpf appear to organise better than those in which DAPT was applied from 48 hpf onwards (Fig. 5G-M), indicating that the ability to organise correlates with the presence of Müller glia in the cultures [shown with GFAP staining (Fig. 5A-F)].

## DISCUSSION

Here, we present a novel model for analysing the cellular and molecular mechanisms governing the cellular interactions that drive lamination in the retina during development. We show that, after disaggregation, dissociated zebrafish retinal progenitors reaggregate quickly; within only 48 h in culture, they are able to organise themselves into layers. With the aid of the SoFa1 line, simple analysis of this layering can be easily and reliably quantified. Using this model, we have begun to investigate the mechanisms of the cellular interactions that drive layer formation in this system, and report here on the relative importance of RPE cells and Müller glial cells in this process.

Our aggregates organise with RPE in the centre, next to photoreceptors and bipolar cells, next to horizontal and amacrine cells, with RGCs on the outside. This normal progression of layers is apparently inverted with respect to the retina *in situ*, where the RPE is the outer cell layer and the inner layer comprises RGCs. Such inside-out organisation was also seen in rosettes within the retinospheroids described by Layer and colleagues (Layer et al., 2001, 2002), and such photoreceptor-centred rosettes, surrounded by inner layer cells have frequently also been seen *in vivo* in pathological conditions. This suggests that there is a natural tendency for retinal cells to organise themselves in layers that does not rely on the polarity of the tissue, and that can happen *in vitro* with disaggregated cells.

Layer and colleagues found that when Müller glia or RPE cells, or even media conditioned by these cell types, are added to reaggregated chick retinospheroids, over the course of several days, the aggregates involute and show retinal-like polarity, with photoreceptors on the outside and RGCs towards the centre (Rothermel et al., 1997; Willbold et al., 2000). As we are most interested in the events that lead to the initial laminar arrangements of cell types, we have not looked over these longer terms in our culture system. In our reaggregation cultures, the lamination happens between 24 hpf and 72 hpf, which is exactly when retinal layering normally occurs *in vivo*. Indeed, we show that zebrafish retinal cells dissociated at 72 hpf do not form organised aggregates, suggesting that there is a restricted time window when this process needs to happen. This finding is reminiscent of work in chick retinal reaggregates, which also showed that complete aggregation (Ben-Shaul et al., 1980) and good layering (Rothermel et al., 1997) in reaggregated cultures could only be achieved when starting with young cells.

It is nevertheless interesting that RPE cells find themselves in the centre of these aggregates, considering that these cells are normally found around the outside of the retina *in vivo*. What is the explanation for this? One possibility is that RPE cells act as seeds or pioneers in the lamination process. We found, however, that these aggregates organise in the same manner in the presence or absence of RPE cells. We were only able to eliminate RPE cells at 32 hpf, leaving open the possibility that they may have an organising influence between 24 and 32 hpf. However, as fully disaggregated cells from 32 hpf retinas organise into laminae, the simplest explanation is that RPE cells are not essential neither for the ability of the neural cells to organise into layers nor for the central to peripheral order of these layers.

The differential adhesion hypothesis model of cellular organisation posits that cells in an aggregate will laminate by minimising their interfacial free energies (Steinberg, 1970, 2007; Foty and Steinberg, 2005). Cells with the strongest adhesions to each other in such aggregates move to the centre whereas cells with weaker adhesions sit further out in the cultures. Recently, it has been shown that cell-cell surface tensions rather than simple adhesion may also drive lamination in tissues (Maître et al., 2015). It would, we feel, be very interesting to investigate in our aggregates how much of a role these physical factors play in retinal lamination. For example, the differential adhesion hypothesis would suggest the strongest adhesions are between RPE cells and the next strongest are between photoreceptors and/or bipolar cells, which occupy the centre of the aggregates when the RPE is removed. These possibilities can be tested using new advances in micro-physical measurements of tension (Puech et al., 2006) and adhesion (Maître et al., 2012).

Previous work from this laboratory has shown that Müller glial cells are among the last cell generated during zebrafish retinogenesis and that the generation of Müller glia is particularly sensitive to blockers of the Notch pathway during this period (MacDonald et al., 2015). The γ-secretase inhibitor DAPT specifically inhibits the Notch pathway, and if applied at 45-48 hpf, completely blocks the formation of Müller glia *in vivo*. Yet in the complete absence of Müller glia *in vivo* in zebrafish, a normally organised retina forms (Randlett et al., 2013; MacDonald et al., 2015). The result reported in this paper, namely that lamination is significantly impaired by the absence of Müller glia in zebrafish organoids, therefore suggests that mechanisms operating *in vivo* but not *in vitro* can compensate for the absence of Müller glia in zebrafish. The possibility that this phenotype is due to other effects of DAPT on retinal lamination cannot be completely ruled out, but the strong correlation between the effects on lamination and Müller glia differentiation suggest that it is the Müller glia themselves that are crucial. Interestingly, in this regard, mouse retinas treated with an antagonist of BMP to block Müller glia differentiation have disrupted lamination and formation of rosettes (Ueki et al., 2015), suggesting that Müller glia may also have a more crucial role in retinal lamination in mammals. The apicobasal polarity of the native neuroepithelium is badly degraded, if not completely destroyed, in the disaggregation-reaggregation process. *In vivo* in zebrafish, cells may be able to sense this gradient and organise themselves along it. Indeed, the native optic cup is a pseudostratified epithelium in which all retinal progenitor cells extend across the entire apicobasal axis; this structure may provide a polarised and oriented substrate for cell migration. In the zebrafish organoids, as in the mouse retina, Müller glia might take on some important role in establishing neuroepithelial conditions. Another possibility relates to the provision of tensile strength to the retina by Müller glia (MacDonald et al., 2015), which is lost when these cells are dissociated but re-established as Müller glia differentiate.

The work of Sasai and colleagues, who generated well-laminated retinal structures starting from mouse and human stem cells (Eiraku et al., 2011; Eiraku and Sasai, 2011), has been particularly exciting for the field of intrinsic tissue organisation. Human organoids of various tissues provide a model whereby one can study developmental mechanisms and diseases that are specific to humans, so one may wonder why it is useful to turn to organoids to study development in a model system such as zebrafish where it is possible to examine retinal lamination *in vivo*. Although the retinal organoids from the Sasai laboratory show that it is not necessary to have a whole embryo to grow an organised tissue, it is clear that these stem cell-derived organoids laminate in the context of a great deal of early pattern that develops in these complex systems, such as the apicobasal cues, patterned extracellular matrix and localised signalling molecules. Thus, the mechanisms at play in these stem cell-derived organoids may be almost as complex as those in the tissues *in vivo*, so it is useful to work on a simplified system. *In vivo* studies in zebrafish and mice have revealed that each cell type in the retina can be eliminated and the remainder of the cells are able to laminate in the correct order (Green et al., 2003; Randlett et al., 2013). In our reaggregated cultures, we provide neither a substrate nor any extracellular matrix with which the cells can interact. This means that the cells must interact with each other in order to sort themselves; their ability to sort themselves into rough layers in the absence of exogenous pattern should help us to identify the molecular and cellular mechanisms involved in the cell-cell interactions at play during retinal lamination.

## MATERIALS AND METHODS

### Animals and transgenic lines

All zebrafish lines were maintained and bred at 26.5°C. Embryos were raised at 28.5°C or 32°C in embryo medium and staged in hours post fertilisation (hpf) using morphological features as described previously (Kimmel et al., 1995). Some embryos were treated with 0.003% phenylthiourea (PTU, Sigma) from 8 hpf onwards to prevent pigment formation. All embryos were anaesthetised with 0.04% MS-222 (Sigma) prior to dissection. All animal work was approved by the Local Ethical Review Committee at the University of Cambridge and performed under the UK Home Office license PPL 80/2198.

Transgenic lines Ptf1a:cytGFP (Godinho et al., 2005), Crx:gapCFP (Almeida et al., 2014) and GFAP:GFP (Bernardos and Raymond, 2006), and the polytransgenic SoFa1 line (Atoh7:gapRFP/Ptf1a:cytGFP/Crx:gapCFP) (Almeida et al., 2014) have all been previously described. Ptf1a:cytGFP/Crx:gapCFP embryos were obtained by crossing homozygous Ptf1a:cytGFP and Crx:gapCFP lines.

### Dissection and dissociation of zebrafish retinas

Embryos (24 hpf or 32 hpf) were anaesthetised and transferred to Ca^2+^-free dissecting medium [116.6 mM NaCl, 0.67 mM KCl, 4.62 mM Tris; 0.4 mM EDTA (pH 7.8) supplemented with 100 μg/ml of heparin and 0.04% MS-222] for retinal dissection. Twenty retinas per condition were collected in fresh dissecting medium in a glass well dish and kept on ice. Retinas were allowed to come to room temperature and incubated with 0.25% Trypsin-EDTA (Sigma) for 12 min. After gentle removal of Trypsin-EDTA, fresh dissecting medium was replaced and retinas dissociated by gentle trituration using a glass fire-polished Pasteur pipette, followed by more vigorous trituration with a P200 pipette until a single cell suspension was achieved. Cells were collected in L-15 supplemented with 3% FBS and centrifuged at 300 ***g*** for 7 min. After gentle removal of 75% of the supernatant, cells were washed once more under the same conditions and re-suspended in the required volume for immediate seeding.

### Cell counting, viability and cluster analysis

Cells were counted, and percentage viability and cluster sizes were calculated using the LUNA-FL Dual Fluorescence Cell Counter (Logos Biosystems). Cells in suspension were mixed with an Acridine Orange/Propidium Iodide mix (Logos Biosystems) and analysed in fluorescence mode.

### Cell culture

Agarose dishes were prepared and cast from 35-well or modified 15-well (cut to size) PDMS moulds (Microtissues) as previously described (Napolitano et al., 2007) using UltraPure LMP Agarose (Invitrogen) and equilibrated with L-15 supplemented with 1% PSF (Thermo Fisher Scientific) within 4-well culture plates (Nunclon). Cells were seeded in a drop-wise manner as a volume of 75 µl into 35-well dishes or of 35 µl into modified 15-well agarose dishes. Cells were allowed 15-20 min to settle before 750 µl culture medium was added via the medium exchange ports. Culture medium consisted of L-15 supplemented with 10% Embryo Extract (https://zfin.org/zf_info/zfbook/chapt6.html), 3% FBS (Thermo Fisher Scientific), 2% N2 (Thermo Fisher Scientific), 1% PTU (Sigma) and 1% PSF. Cells were incubated at 28.5°C for 48 h before aggregates were harvested for analysis.

### Drug application

For the Müller glia experiments, cells were incubated with 25 μM DAPT (Sigma) or the equivalent volume of DMSO starting from the equivalent time of 45-48 hpf, or 63 hpf.

For the R-Cognin experiments, cells were incubated with 5, 50, 100 or 200 μM PACMA31 (Sigma) or the highest equivalent volume of DMSO from the time of cell seeding onwards.

### Harvesting of aggregates, fixation and mounting

Aggregates were fixed with 4% PFA for 20 min at room temperature followed by 3×5 min washes with PBS and collection by gentle downward flushing action using a P200 pipette. Aggregates were mounted in VectorShield mounting medium with DAPI (Vector Laboratories), surrounded by a reinforcement ring between a microscope slide and a 13 mm round coverslip.

### Immunostaining

Aggregates were fixed in 4% PFA for 15 min at room temperature followed by a 10 min wash with 0.1% PBT and then PBS. Aggregates were then incubated overnight at 4°C with primary antibodies. Aggregates were washed for 10 min with 0.05% PBT and incubated for 2 h at room temperature with the secondary antibodies together with DAPI (1:1000). Aggregates were washed for 10 min with PBT then 10 min with PBS before mounting. Aggregates stained with Zn5 were additionally blocked for 20 min at room temperature (10% HIGS, 1% BSA, 0.5% Triton in 1× PBS) before the primary antibody incubation. Antibodies used were mouse anti-Zn5 (1:100; ZIRC) and mouse anti-GFAP (1:100 zrf1; ZIRC).

### Confocal image acquisition and analysis

Aggregates were imaged under an oil-immersion 60× objective (NA=1.30) using an inverted laser-scanning confocal microscope (Olympus FV1000). Images were acquired for seven *z*-slices at the centre of each aggregate at 1μm optical sections using the same settings throughout: 1024×1024 resolution, 12.5 µs/pixel scanning speed. Data were acquired using Olympus FV1000 software and analysed using Volocity Software (Perkin Elmer).

### Analysis of organisation by isocontour fluorescence profiling

The central most section of each aggregate was analysed using custom-made Matlab scripts. The geometry of the aggregate was determined from the DAPI image via automatic segmentation [active contour (Chan-Vese) and morphological operators based] (Chan and Vese, 2001) or manual segmentation. The manual method allowed the user to correct for easily recognised artefacts such as dead cells on the outside of the aggregate, which no longer express fluorescent protein.

The fluorescence inside the aggregate was characterised by the intensity profile obtained via averaging the pixel intensities in concentric bands of width w=5 pixels. We examined two ways to construct the fluorescent profiles: (1) averaging pixel intensities in circular crowns around the centroid of the aggregate; (2) averaging the intensities in bands of equal width starting from the periphery (outline of the aggregate) to the centre. Although the results are similar for both cases, we adopted the latter method as it is more robust to the variation of aggregate shape.

In order to be able to compare sets of profiles from different experiments, the fluorescent intensity profile was normalised such that its integral is 1 and the distances to the centre were rescaled between 0 and 100 radial units. Subsequently, the cumulative profiles were computed (ECDF plot), depicting how the distribution of fluorescence for each FP differs from a random distribution. From this, we used trapezoidal numerical integration to find the area beneath each curve and then subtracted that of the Ptf1a:cytGFP curve from the Crx:gapCFP curve to calculate the area between the two curves.
